# Eliminating sedimentation for the treatment of chronic pelvic pain syndrome

**DOI:** 10.3892/etm.2013.982

**Published:** 2013-02-27

**Authors:** ZHONGMING SUN, YANZHONG BAO

**Affiliations:** Department of Reproductive Medicine, Zhejiang Provincial Hospital of Integrated Chinese and Western Medicine, Xiacheng, Hangzhou, Zhejiang 310003, P.R. China

**Keywords:** chronic pelvic pain syndrome, prostate Qi-concentrated single-finger manipulation, eliminating sedimentation, NIH-CPSI score

## Abstract

The aim of this study was to evaluate the curative effects of eliminating sedimentation inside the prostate via manipulation for the treatment of chronic pelvic pain syndrome (CPPS) using the National Institutes of Health (NIH)-chronic prostatitis symptom index (CPSI) scores. According to the prostatitis classification standard of the NIH, 721 patients with CPPS were divided into groups IIIA and IIIB by prostatic fluid routine examination (EPSRt) and treated using manipulation. The treatment was performed once per 3 days for 3–5 min and 10 treatments were considered to be a period. The EPSRt and NIH-CPSI scores were tested before and at the end of each period following treatment. After 3 treatment periods, the effectiveness and total effectiveness rates of the IIIA group were 72.3 and 15.9%, respectively and those of the IIIB group were 71.8 and 16.3%, respectively. Statistical analysis showed no significant differences between the curative effects in the two groups (P>0.05). The NIH-CPSI scores of the two groups were significantly improved following each treatment period (P<0.01). Eliminating sedimentation using manipulation dispersed the blockage, discharged the turbidity and cleared the gland, leading to the elimination of sedimentation and the relief of sinus hyperemia around the prostate, which significantly improved the clinical symptoms of CPPS and the quality of life of the patients.

## Introduction

Chronic prostatitis has a high incidence; its prevalence rate has been reported to be 9%, similar to that of ischemic heart disease or diabetes (1). It is estimated that the global incidence is 9–14% (2). According to the Classification Criteria of the United States National Institutes of Health (NIH) (3), prostatitis syndromes are divided into 4 categories: type I is acute bacterial prostatitis, type II is chronic bacterial prostatitis, type III is chronic non-bacterial prostatitis/chronic pelvic pain syndrome (CNP/CPPS) and type IV is asymptomatic inflammatory prostatitis. CNP/CPPS may also be divided into inflammatory [IIIA, white blood cell (WBC) >10/high power field (HPF) in expressed prostatic secretion (EPS)] and non-inflammatory (IIIB, WBC ≤10/HPF in EPS) categories (4). CPPS is the most common form of chronic prostatitis, a frequently occurring disease in males with an unknown etiological mechanism, complex clinical symptoms, specific anatomical position and clinically recurrent attacks.

Antibiotics, particularly quinolones, affect the cytokine activity of the immune response, specifically by regulating the expression of IL-6 and IL-8 and decreasing the cytokine levels in the prostate fluid of chronic prostatitis patients. Antibiotics also have analgesic effects, which have been reported to alleviate the pain symptoms of chronic prostatitis (5–9). However, there have been contrary studies (10) in which antibiotics were not observed to be the preferred therapy. In patients treated with antibiotics, it was observed that the patient-perceived symptom improvement rate, symptom score improvement rate and prostate fluid routine WBC reduction rate were not statistically significantly improved compared with those of the control group. In particular, the associated pain, which was not treated satisfactorily for a long period, caused the patients to lose confidence and even led to suicidal thoughts.

At present there is no definitively effective treatment and numerous patients experience various degrees of sexual dysfunction, neuropsychiatric symptoms, fatigue, insomnia and mood disorders, and semen quality is seriously affected in certain patients. The condition is a cause of infertility and seriously affects the quality of life and work. Currently, treatments such as Western or Chinese medicine and physical therapy do not have good efficacy and if patients discontinue their medication for a few days, the symptoms may return to their original status, without signs of improvement. Certain patients even consider that the symptoms are aggravated by medication. We propose that the reasons for the above lie in the fact that the therapy is unable to clear the accumulation of inflammatory metabolites from the prostatic tube, acini and interstitial region. Long-term blockage, hardening and tension in the local gland duct is likely to generate a variety of symptoms and since these factors are not readily relieved by drug therapy or the autologous system, the treatment is invalid. It has been suggested that treatment should eliminate these factors, as in the Western therapy theory, of ‘operating on an ulcer to clean a toxin’. Only in this way may the treatment provide instant results.

In order to improve the treatment of CPPS, we combined the prostatic anatomy of Western medicine with Chinese acupuncture principles and techniques, to design a one-finger manipulation method for clearing the blockage. This may eliminate the deposits of inflammatory metabolic products from the prostate, in a similar manner to Western incision methods, improve the local metabolism and circulation, rapidly eliminate inflammatory products and improve the symptoms. The clinical application of this approach achieved stable curative effects and satisfactory results.

## Subjects and methods

### Clinical data

Between January 2008 and March 2012, 721 cases of CPPS, comprising male outpatients from the Department of Reproductive Medicine, Zhejiang Provincial Hospital of Integrated Chinese and Western Medicine (Hangzhou, China), including 307 unmarried and 414 married patients, were treated and observed. All the patients had a sexual history and the course of the condition was 5–183 months, with an average of 28.3 months. The patients all received antibiotics, α receptor blockers, antiphlogistic analgesics and drugs, which were ineffective. Therefore, patients were treated with manipulation. The oldest patient was 63 years old, the youngest was 17 years old, and the average age was 32.6 years old. The symptoms were as follows: 320 cases of perineal pain, 291 of perianal pain, 340 of lower abdomen and groin area aches, 298 of penile pain, 251 of double inner thigh pain, 293 of urinary frequency, 233 of more-frequent nocturia, 121 of urine bifurcation, 302 of delayed urination, 293 of neurasthenia, 353 of sleep disorders, 387 of sexual dysfunction, 373 of memory decline, 391 of fatigue, 149 of testicular pain, 187 of lumbosacral pain, 118 of suprapubic pain, 194 of urinary pain or burning sensations, 43 of ejaculatory pain, 238 of incomplete urination, 238 of wet scrotum and 229 of increased dreams. The rectal digital examination results were as follows: 453 cases of unsmoothly surfaced prostate, 463 of texture hardening, 393 of swelling and 565 of nodosity. In the prostate fluid routine examination, the leukocyte counts were >3–40/HPF, the lecithin corpuscle density was reduced and the EPS bacterial culture results of all cases (Meares-Stamey two-cup method) (3) were negative, consistent with the diagnostic standard of NIH-CPPS. Ultrasound examination prior to the treatment showed inhomogeneous echo or calcification; no echo area around the prostate peripheral area; or no clear echo from the prostate film. The present study was conducted in accordance with the Declaration of Helsinki and with approval from the Ethics Committee of Zhejiang Provincial Hospital of Integrated Chinese and Western Medicine. Written informed consent was obtained from all participants.

The patients were divided into two groups according to the EPS routine examination results: group IIIA (inflammatory group), 440 cases (EPS WBC >10/HPF) and group IIIB (non-inflammatory group), 281 cases (EPS WBC ≤10/HPF). The average ages were 32.2 and 32.8 years and the average durations were 28.2 and 28.5 months in groups IIIA and IIIB, respectively. According to the chronic prostatitis symptom index (NIH-CPSI) scoring method (11), the scores in the two groups were 26.79 and 24.59, respectively, with no significant difference between them (P>0.05).

### Treatment methods

The treatment method was based on the prostate massage method (12). By combining traditional Chinese medicine acupuncture principles and techniques. we designed the prostate manipulation method, detailed as follows: i) The physician must understand traditional Chinese medical theory and knowledge and have mastered the basic skills and techniques of massage, particularly one-finger manipulation massage (13–18). ii) The patient first evacuated their bowels by defecation, then the physician pulled back the foreskin of the patient’s penis to completely expose the urethra and sterilized disposable film gloves were used to cover the whole scrotum and penis and accept the prostatic discharge. The patient adopted a knee-chest position while kneeling down on a couch with the arms on the sides of the head and the waist angled straight downward. The angle between the lumbar and thigh was 75–85° and the anus formed an angle of ∼45° with the couch surface. iii) The physician wore sterilized medical disposable latex gloves or disposable PE gloves and stood upright to the right (left), parallel to the patient. PPI-iodine was used for the disinfection of the anus and surrounding area. After lubricating the left (right)-hand finger with a disinfected medical cotton ball or vaseline paraffin oil, the physician then lubricated the patient’s anus with paraffin oil cotton or vaseline, using gentle massage to facilitate acceptance by the patient. The right (left)-hand finger gently contacted the patients’ external anal aperture and was inserted into the rectum along the rectal tube, until it touched the prostate. iv) Through digital rectal examination, the prostate size, texture, presence of nodules, tenderness, swelling, elasticity and degree of smoothness were evaluated (19). v) When performing one-finger manipulation, the central sulcus was set as the center line, and standard manipulation techniques from right (left) to left (right), such as pointing, rolling, rubbing and pushing, were used. The massaging procedure was carried out in segmental order, with each section treated 3–4 times. The pressure applied was flexible, and varied in power and strength according to the lesion texture. The strength and technique applied were selected according to the observed status of the patient. For instance, if the finger pulp was able to touch the end section of the prostate, then the hook-push method was applied. In certain patients, the hooking-push of the prostate was carried out along the longitudinal axis, by compressing and sliding forward and backward, which was more conducive to prostate drainage. In the central sulcus, the finger pulp applied point-pressure and linear-pressure from the distal to proximal points, with the same method repeated on the other side. If necessary, the physician supported the lower back and abdominal parts of the patient with the left (right) hand to adjust the patients’ posture if pain was experienced, while observing the patient’s behavior and expression and talking with the patient to relieve his pain. When the patient felt an effusion, repeated pushing and pressing once or twice was used and the patient was asked to kneel straight. The physician used their left (right) hand to squeeze, pinch, rub and press homeopathically forward from the penis and scrotum root, which forced the overflowing prostatic secretions to flow into the film glove. These secretions were then sent for testing. If the drainage method was used, photographic images of the secretions were captured to enable changes in the quantity and properties of the prostate discharge during the whole process of manipulation to be observed. vi) Following the manipulation, clockwise (counterclockwise) point-pushing with the finger pulp was used to treat prostate nodules, stiffness, swelling, tenderness and induction points, for the facilitation of improved discharge of the local gland duct sediment, while paying attention to the patient’s response. When necessary, the left (right) hand performed corresponding matching massage in the lower abdomen and perineal areas while the right (left) finger manipulated. For example, two-finger manipulation: the exterior and interior fingers corresponded to each other, separated by related tissues in the abdomen and perineum, while they pushed; palm-finger manipulation: using the exterior part of the palm, the finger was pushed around, setting the palm center to the settling point, with the hand pushing around this center; fist-finger manipulation: the finger pulp pointed at the corresponding glands or sensitive points, then the fist was used to move the body forwards and backwards. During the use of these methods, sustainable clamping pressure was applied to the tissues to strengthen the discharge capacity of the continuous secretion. vii) Finally, the physician slowly withdrew the finger, stopping for 3–5 sec when it was removed from the anus. viii) During the entire drainage therapy, attention was paid to the patient’s tolerance and care was taken to apply moderate strength, a stable force and safe methods, and to avoid injury to the rectum.

### Observation indices

The manipulation was performed once every 3 days and during the treatment, tobacco, alcohol and spicy food were prohibited and only moderate sexual activity was recommended. Treatment periods were set as 30 days and at the end of each treatment period, follow-up was performed to assess the NIH-CPSI score (11) and carry out routine EPS examination. After 3 periods, the therapeutic effects were evaluated. Prior to and following the treatment, routine prostate, liver function, blood, urine, electrocardiogram and adverse reactions were evaluated.

### Efficacy standard

The EPSRt WBC count scoring criteria used in the two groups was as follows: 0 points, WBC count <10/HPF; 1 point, 10–20/HPF; 2 points, 20–50/HPF; 3 points, >50/HPF. The efficacy criteria were: significantly effective, NIH-CPSI score reduced by ≥10 points compared with that before treatment and EPS WBC count score reduced by ≥2 points compared with normal or that before treatment; effective, NIH-CPSI score reduced by ≥5 points compared with that before treatment and EPS WBC count score reduced by ≥1 point compared with that before treatment; invalid, NIH-CPSI score reduced by <5 compared with that before treatment or EPS WBC count score reduced by <1 compared with that before treatment.

### Statistical analysis

SPSS 15.0 software was used to carry out the statistical analysis and the measurement data were expressed as the mean ± SD. Comparisons of NIH-CPSI score and WBC count score in EPSRt used the paired t-test and the rank-sum test was used for comparisons between the two groups. P<0.05 was considered to indicate a statistically significant result.

## Results

### Treatment results

Following three treatment periods, the effectiveness rate in the IIIA group was 72.3%, with an efficiency of 15.9%, while those of the IIIB group were 71.8 and 16.3%, respectively. Through statistical analysis, the effects in the two groups were not significantly different (P>0.05). The NIH-CPSI and EPSRt WBC count scores before and after treatment are shown in Table I. Following each treatment, all NIH-CPSI scores of the two groups were significantly improved (P<0.01; P<0.05). The group IIIA WBC count score was reduced in the first and third course, although no clear changes were observed following the second course (Table I). Following the treatment, ultrasound examination revealed that the prostate inhomogeneous echo, film anechoic and anechoic regions surrounding the prostate had disappeared in 306 cases.

During treatment, 493 cases experienced a small amount of abdominal perineal pleasure, while in the first 3–5 manipulations, 247 cases appeared to suffer from increased flatulence or defecation frequency, 35 had small amounts of blood in their urine, 25 ejaculated a brown liquid and 95 had thick byssine urine. The remaining patients had no serious adverse reactions. The liver and kidney function, blood, urine and electrocardiogram had no abnormal changes.

## Discussion

In 1998, the International Prostatitis Collaborative Network Conference confirmed the chronic prostatitis syndrome classification standard and the NIH-CPSI scoring system presented by the NIH in 1995 (3), which are the current gold standard evaluation methods for the diagnosis and treatment of chronic prostatitis.

Chronic prostatitis (CP)/CPPS comprises a group of pelvic pain symptoms and voiding syndromes which are difficult to define and cause anxiety (19). The potential factors which may cause prostate congestion, such as frequent sexual intercourse, masturbation, alcohol, spicy food, cold and extended pressure to the perineum, possibly induce CPPS or exacerbate the symptoms. However the exact etiology and pathogenesis of CPPS are extremely complex, including urine reflux (chemical prostatitis), autonomic dysfunction, immune-related disorders, bacterial infection and other factors (20–23). Treatments also vary greatly, particularly empirical clinical therapies, and in certain patients are not effective (24). Treatments using α receptor blockers, antibiotics, plant preparations, anti-inflammatory drugs and antidepressants are commonly used, but have no definite effect. Certain studies have reported that treatment with α receptor blockers significantly mitigated the difficult to treat symptoms which cause damage to the lower urinary tract, but had no clear effects on pelvic pain and other symptoms. In the cases where α blockers or antibiotic treatments were ineffective, i.e. refractory CP/CPPS, a single drug therapy was unable to produce a satisfactory curative effect, even if administered for an adequate period (24). It has been suggested that, even for newly diagnosed cases, α receptor blockers may have no positive effect. For refractory CP/CPPS, orally administered α receptor blockers or antibiotics have been reported to have similar effects to a placebo (24,25). Terasaki *et al* proposed the intrapelvic venous congestion (IVC) etiology of prostate pain and designated it IVCS (26).

Therefore, the treatment of this disease remains controversial. The most feasible treatment scheme is: i) direct relief of pain symptoms; ii) diagnostic follow-up; and iii) pilot etiological therapy (27).

In traditional Chinese medicine, this condition belongs to the categories of muddy semen, consumptive micturition disorders, lumbago due to deficiency of the kidney and other fields. Long-term disease often leads to deficiency of the kidney and difficulty in recovering. Anatomically, the prostate is located at an interchange position of Ren, Du and Chong and is the center of Qi and blood circulation. Prostatitic swelling, nodule formation and fibrosis cause blockage of the meridian or even stagnation, as observed from the principle of veins and arteries and causes feelings of discomfort such as swelling and pain.

**Figure 1:**
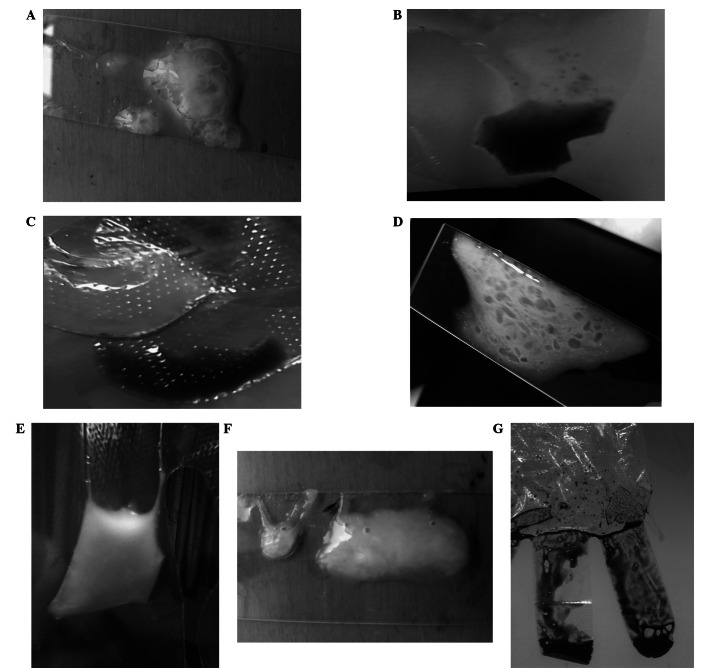
Examples of prostatic discharge during treatment. (A) Thick phlegm-like gray jelly-like fluid; (B) rusty blood clot; (C) green jelly-like fluid; (D) gray jelly-like fluid; (E) light yellow jelly-like fluid; (F) creamy white jelly-like fluid; (G) pitch-like blood clot.

With regard to the pathological mechanism, ‘blood stasis’ is the basic pathogenesis (28), mainly caused by phlegm, heat and blood. According to the therapeutic concept of ‘fluency is the basis’ in traditional Chinese medicine, among the patients who had experienced no clear effects following long-term treatment, single finger manipulation was able to apply pressure to soften the blockage, eliminate the turbidity and cause the sediment to be secreted. Clearing the gland may accelerate the removal of pathogenic microorganisms and toxins, comparable to a debridement and drainage effect, promoting the abrogation of inflammation, improving gland duct drainage, reducing sympathetic excitability, relieving urethral smooth muscle resistance and ensuring the prostate catheter drainage is unobstructed, thereby eliminating aches and swellings due to the blockage and the multifactoral suffering caused by inflammatory lesions. Following the prostatic massage procedure, the majority of patients discharged a large amount of sediment, turbid fluid, coagulation and blood, with yellowish, green, gray and rusty colors (Fig. 1).

**Figure 2:**
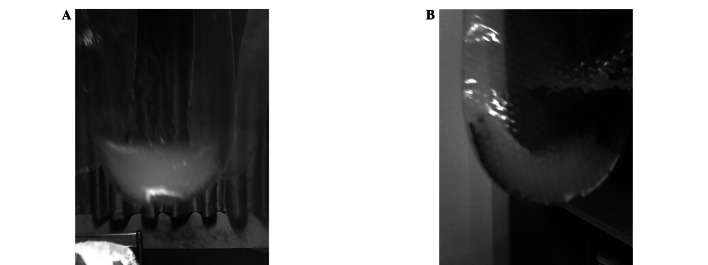
Examples of prostatic discharge following treatment from different patients. (A and B) Wuliangye alcohol-like prostatic fluids.

Certain patients felt relaxed and their symptoms immediately disappeared. The discharge was to a certain degree like thick pus or nasal mucus, with a jelly-like fluid containing a variety of suspended foreign substances. These substances were routinely of unknown composition and were difficult to identify. Finally, when all the prostate nodules had been cleared, the glandular tubes were immediately unobstructed and the sediment was discharged, inflammation disappeared and the discharged prostate fluid was clear, similar in appearance to the alcohol (Fig. 2). It appeared to be light blue in the glass dish. There was no clear discomfort. The symptoms, including perineal pain, lower abdominal pain, waist and rib soreness, headaches, dizziness, wet scrotum, increased dreams and dysphoria, were reduced significantly or even disappeared following the massage, while mental status and sexual dysfunction also improved significantly. However, symptoms may recur if the patients develop an unhealthy lifestyle. Therefore healthy living and working behavior should be enforced, and repeated, continuous and regular manipulation should be maintained to eradicate the disease. Practical observations suggest that CPPS is usually repeated focal inflammation rather than prostatitis affecting the whole prostate, where the prostate gland is partially mechanically obstructed and pressing on the prostatic stroma or prostate surrounding area. Therefore, the symptoms and prostate palpation findings of the patients are changeable, and are correlated with life and work factors.

The results showed that, following each treatment period, the NIH-CPSI scores for urinary pain and discomfort symptoms and quality of life in the two groups were improved significantly (P<0.01), indicating that the prostate massage improves the clinical symptoms of CPPS. When comparing the EPS WBC count scores of the IIIA group before and after treatment, the changes following the 2nd course were not clear, while the scores following the 1st and the last course were reduced.

In the present study, the total effective rate in the 721 cases following 3 courses of treatment was 72.1% and the average obvious effective rate was 16.1%, giving a total effective rate of 88.2%, which was a satisfactory effect. No serious adverse reactions occurred in the course of the treatment. During treatment, it is recommended that attention is paid to diet, optimistic spirit, moderate work with rest, proper exercise and sweating, early sleep and a regular sex life (avoiding congestion and stagnation), which are likely to contribute to the rehabilitation from this disease.

## Figures and Tables

**Table I:** Comparison of the NIH-CPSI and EPSRt WBC count scores of the two groups (mean ± S).

Group	Period	Pain	Urine symptoms	Life quality	Total score	WBC count score
IIIA (n=40)	Before	10.53±3.31	7.56±2.29	9.35±2.87	26.79±8.31	2.56±0.87
	1	7.38±4.02^a^	5.96±2.74^a^	7.82±3.21^a^	21.79±7.39^a^	1.79±0.73^a^
	2	6.10±4.36^a^	4.48±2.35^a^	6.49±3.31^a^	17.98±7.89^a^	1.51±0.83
	3	3.83±1.59^a^	2.19±1.12^a^	3.23±1.37^a^	9.13±3.79^a^	1.03±1.11^b^
IIIB (n=281)	Before	9.93±3.12	6.96±2.37	8.93±3.25	24.99±8.73	
	1	7.04±2.13^a^	4.93±1.39^ad^	6.83±2.23^ad^	19.73±5.69^ac^	
	2	5.77±2.27^a^	3.39±1.41^a^^d^	5.39±2.14^ac^	14.91±6.15^ac^	
	3	2.19±0.93^ac^	1.93±1.11^a^	2.91±1.31^a^	7.53±3.21^ac^	

a P<0.01,

b P<0.05, vs. before treatment.

c P<0.01,

d P<0.05, vs. after treatment in each group. NIH-CPSI, National Institutes of Health-chronic prostatitis symptom index, EPSRt, prostatic fluid routine examination; WBC, white blood cell.
